# Air-fed cold atmospheric plasma device as a safe and effective anti-SARS-CoV-2 air filter

**DOI:** 10.1038/s41598-026-36088-y

**Published:** 2026-01-12

**Authors:** Fei Cao, An Yan, Qingnan Xu, Qiujie Fang, Xiao Chen, Shuang Xue, Longfei Qie, Jie Fang, Gbenga A. Martins, Ye Lu, Jamoliddin Razzokov, Ruixue Wang, Guiqiang Wang, Zhitong Chen

**Affiliations:** 1 Paul C. Lauterbur Research Center for Biomedical Imaging, Institute of Biomedical and Health Engineering, Shenzhen Institutes of Advanced Technology, Chinese Academy of Sciences, Shenzhen, 518055 China; 2 College of Mechanical and Electrical Engineering, Beijing University of Chemical Technology, Beijing, 100029 China; 3 Zhejiang Provincial Laboratory of Experimental Animals & Nonclinical Laboratory Studies, Hangzhou Medical College, Hangzhou, 310059 China; 4 Department of Environmental Health, Zhejiang Provincial Center for Disease Control and Prevention, Hangzhou, 310051 China; 5 Institute of Fundamental and Applied Research, National Research University TIIAME, Kori Niyoziy 39, Tashkent, 100000 Uzbekistan; 6 Department of Chemical Engineering and Biotechnology, Karshi State Technical University, Mustaqillik Avenue Street 225, Karshi, 180100 Kashkadarya Uzbekistan; 7 Department of Biotechnology, Tashkent State Technical University, Universitet 2, Tashkent, 100095 Uzbekistan; 8 Department of Infectious Disease, Center for Liver Disease, Peking University First Hospital, Beijing, 100034 China; 9 Department of Infectious Disease, Peking University International Hospital, Beijing, 102206 China; 10 Advanced Therapeutic Center, National Innovation Center for Advanced Medical Devices, Shenzhen, 518100 China; 11 State Key Laboratory of Biomedical Imaging Science and System, Chinese Academy of Sciences, Shenzhen, 518055 China

**Keywords:** Cold atmospheric plasma, SARS-CoV-2 disinfection, Electron density, Morphological spikes, Safety evaluation, Diseases, Health care, Medical research, Microbiology, Physics

## Abstract

Cold atmospheric plasma (CAP) displays antiviral properties, and its efficacy and safety in daily use are subject to full elucidation. In this work, we develop an air-fed CAP device, by inhaling and filtering, to directly inactivate SARS-CoV-2 in the air. The plasma shows a high intensity with reactive nitrogen and oxygen species in optical emission, with plasma electron temperature and density of 0.31 eV and 3.25 × 10^17^ m^-3^, respectively, confirming a strong discharge for SARS-CoV-2 disinfection. A qualitative analysis of the device’s discharge process is conducted using a two-dimensional fluid model. The disinfection results show that the typical SARS-CoV-2 morphological spikes disappear after plasma treatment, and the proteosomes of the SARS-CoV-2 virus change. In addition, we conduct experiments on rats to evaluate the safety of the air plasma device in daily use. Rats’ behavior, body weight, food consumption, organ histopathology, and blood biochemical indicators after exposure to this device are evaluated. These results demonstrate that under our experimental conditions, this prototype plasma–air circulation system altered the structural integrity of SARS-CoV-2 and did not produce overt acute toxicity in rats over 4 weeks, although some serum chemistry parameters changed and require further evaluation.

**Supplementary Information:**

The online version contains supplementary material available at 10.1038/s41598-026-36088-y.

## Introduction

Coronavirus disease 2019 (COVID-19), an emerging contagious disease caused by severe acute respiratory syndrome coronavirus 2 (SARS-CoV-2), has induced a serious century pandemic^1^. COVID-19 has spread to all continents with multiple epicenters and globally caused around 7 million deaths. Although vaccines, small-molecule agents, antibodies, and certain physical treatments have been shown to assist patients to fight COVID-19, no remedies thoroughly solve the problem so far, inducing high mortality rates, especially in senior groups^2,3^. COVID-19 transmission between people mainly occurs through bioaerosol inhalation or self-inoculation in the mouth and eyes. These two transmission conditions are facilitated in many ways: droplets, direct contact, fomite, aerosols, blood-borne, fecal-oral, and mother-to-child^4^. Therefore, purifying the air is an effective way to prevent the spread of the SARS-CoV-2 virus. Recent progress in plasma has led to the creation of atmospheric and room temperature plasmas, which is cold atmospheric plasma (CAP). CAP has been used for a wide range of biomedical applications, including virus inactivation^5–9^. The efficacy of CAP is because of its components that exhibit favorable behavior for biomedical applications, including electrons, charged particles, reactive oxygen species (ROS), reactive nitrogen species (RNS), free radicals, ultraviolet (UV) photons, molecules, electromagnetic fields, physical forces, and electric fields^10–15^.

Chen et al. employed CAP to inactivate SARS-CoV-2 on various surfaces in UCLA P3 Lab, including plastic, metal, cardboard, basketball composite leather, football leather, and baseball leather^16^. Their results demonstrated the great potential of CAP as a safe and effective means to prevent virus transmission and infections. Ibáñez-Cervantes et al. examined the disinfection capacity of H_2_O_2_ plasma against SARS-CoV-2 and bacteria (Acinetobacter baumannii and Staphylococcus aureus) through N95 masks, and they pointed out that H_2_O_2_ plasma was an efficient way to disinfect N95 masks^17^. In addition, an 80-day clinical trial took place in a hospital to evaluate the CAP lowering the viral load in the COVID-19 room, and results indicated that CAP could decrease coronavirus spread in hospitals and prevent virus transmission^18^. Wang et al. employed cold atmospheric pressure plasma to treat wastewater contaminated with SARS-CoV-2, achieving effective viral inactivation within 180 s^19^. Kurt et al. developed a novel disinfection chamber that utilizes ozone generated by plasma to regularly disinfect non-porous surfaces^20^. Overall, CAP has been demonstrated to be significantly effective in inactivating SARS-CoV-2 in most scenarios.

It’s known that the disruption of the interaction between the receptor-binding domain (RBD) and human angiotensin-converting enzyme 2 (hACE2) can prevent coronavirus infection. Scientists utilized plasma or plasma-activated media to induce spike protein or RNA damage to demonstrate that plasma works for the inactivation of SARS-CoV-2^21,22^. Attri et al. employed molecular dynamic (MD) simulations to elucidate that the C-terminal domain of the SARS-CoV-2 spike protein structure became unstable after plasma oxidation, and binding free energy decreased with plasma-induced oxidation^23^. By disrupting biomacromolecules, plasma-activated water can effectively inactivate bacteria and bacteriophages. Kong et al. demonstrated, using a virus model with the SARS-CoV-2 S protein, that plasma-activated water can reduce the binding of RBD to hACE2^24^. However, there is no direct evidence showing the proteins change of SARS-CoV-2 after plasma treatment.

In this paper, we develop a CAP device with air as the feeding gas. The electrical and optical characterization of plasma were performed. A qualitative analysis of the device’s discharge process was conducted using a two-dimensional fluid model. We utilized transmission electron microscopy (TEM) to characterize morphological changes and proteins denaturation of SARS-CoV-2 after plasma treatment. In addition, systemic evaluation of the safety of the air plasma was conducted through rat experiments. Our study revealed the direct alterations of SARS-CoV-2 by plasma treatment, while evaluating the impact of this air plasma on various biological parameters. This work may provide robust support and guidance for the virus inactivation applications of air plasma.

## Results

### Discharge characteristics

**Fig. 1:**
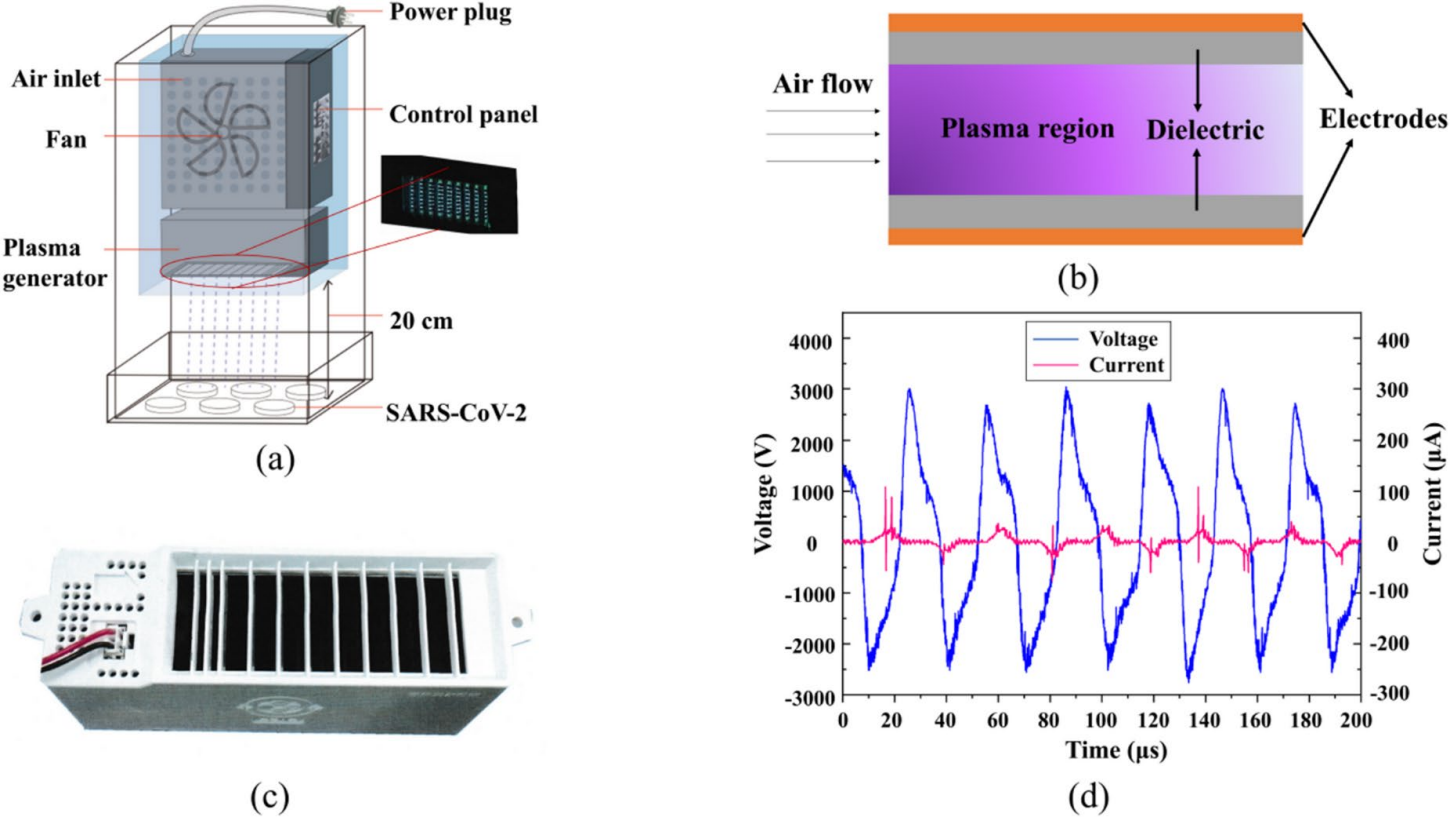
The air plasma device and discharge. (**a**) The schematic diagram of the experimental setup containing air plasma device discharge and SARS-CoV-2 inactivation. (**b**) A schematic illustration of the configuration of a discharge unit. (**c**) A real picture of the plasma device. (**d**) I-V curves of plasma discharge.

Design of the air plasma device includes the power plug, control panel, air inlet, fan, and the plasma generator, which is depicted in Fig. 1a. The cold plasma generator adopted in this study consists of a comb-shaped electrode array, a high-voltage power supply and an isolation housing (isolating the electrode array from the high-voltage power supply). The electrodes are surface metal coatings of 30 μm in thickness on a printed circuit board (PCB). The distance between electrodes is about 3 mm. An schematic illustration of a discharge unit is shown in Fig. 1b. The plasma generation during discharge is shown in the insert of Fig. 1a. Plates containing SARS-CoV-2 virus were put at 20 cm distance below the plasma outlet for the inactivation experiment. A photograph of the device entity is shown in Fig. 1c. Figure 1d indicates I-V curves of the air plasma device. The high-voltage power supply provides alternating high voltage to the comb-shaped electrode array. When a flow of air passes through the comb-shaped electrode array, the charged electrodes ionize the flowing air. Since the voltage of the electrodes is continuously changing, mixed plasma clusters with multiple particle components are generated at different voltages, such as positive ions Ar^+^, N_2_^+^, O_2_^+^, H^+^, and negative ions O^-^, O_2_^-^. The alternating high voltage generated by high-voltage power supply has a peak-to-peak voltage of 5.88 kV and a frequency of 33 kHz, the corresponding discharge current is 123µA (Table 1). The device works in a dielectric barrier corona discharge mode and charge leakage was also assessed (**Supplementary Table ****S1**). Parameters were measured at room temperature and relative humidity of 47%.

**Table 1:** Characterization of the plasma device.

Number	Parameter	Measurement result
1	Operating voltage V_p−p_ (kV)	5.88
2	Operating current I_p_ (µA)	123
3	Operating frequency υ (kHz)	33
4	Breakdown voltage V_p−p_ (kV)	2.53
5	Plasma electron temperature T (eV)	0.31
6	Plasma electron density ($\:\times\:{10}^{17}{m}^{-3}$)	3.25

**Fig. 2:**
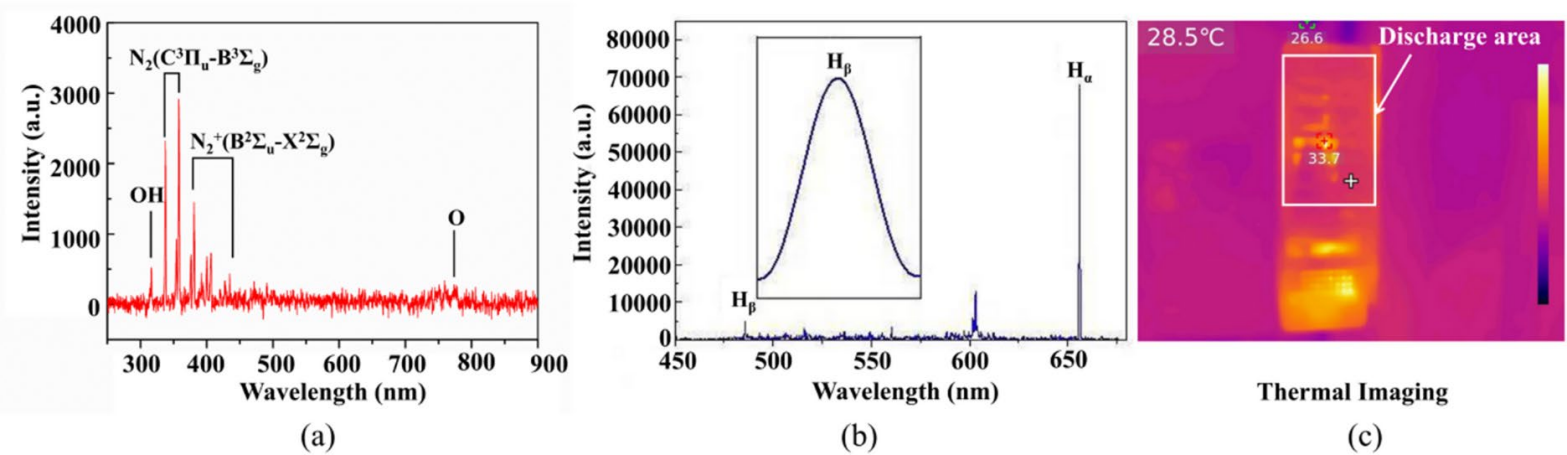
Characteristics of the device. (**a**) The optical emission spectra (OES) of plasma air discharge. (**b**) Plasma emission spectrum of hydrogen discharge in a dark environment. (**c**) Thermal imaging of the device during discharge.

We captured the optical emission spectra (OES) of the device during air discharge, as shown in Fig. 2a. The primary reactive species were marked, with excited nitrogen accounts for the majority, an OH peak and a small amount of atom oxygen. To further investigate discharge characteristics, the device was placed in a chamber filled with hydrogen followed by discharge, which allowed us to capture the plasma emission spectrum in a dark environment. Figure 2b shows the OES of plasma discharge in hydrogen, which were detected by a monochromator with 0.02 nm H_β_ fine spectrum resolution. Atomic levels are broadened and shifted because of the Stark effect, caused by electric micro-fields formed by ions and electrons. Here, we considered the Stark broadening theory of the Balmer lineage of hydrogen atoms, we employed the Inglis-Teller equation to calculate the plasma equivalent electron temperature. The Inglis–Teller equation indicates an approximate relationship between the plasma density and the principal quantum number of the highest atom-bound state derived by David R. Inglis and Edward Teller. The plasma equivalent electron density was calculated by the Boltzmann plot method. The plasma electron temperature and density were 0.31 eV and 3.25 × 10^17^ m^-3^, respectively (Table 1). It indicates that our plasma generator has the potential for sterilization and disinfection with great efficiency. The working temperature of the plasma device was 33.7 ℃ in maximum and 28.5 ℃ in average gas temperature within the discharge region measured by a thermal meter at 20 cm of distance to the air outlet, indicating its user-friendly property (Fig. 2c).

**Table 2:** Boundary conditions of the plasma model.

Boundary	Electrostatic condition	Species condition
AB	Constant potential	NA
	V = 8 kV Fig. 3(b)	
DE/FG	Field continuity	Wall losses
AH/CJ	Constant potential	Continuity
	V = 0 kV	
IJ	Ground	NA
	V = 0 kV	

**Fig. 3:**
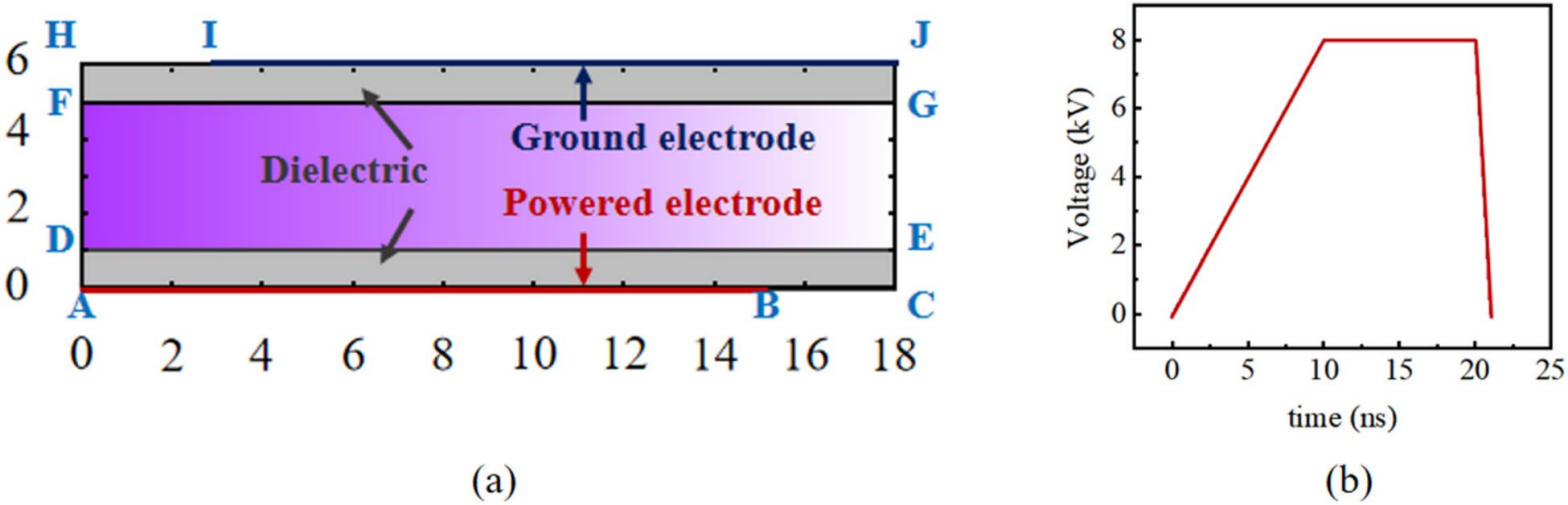
Simulation parameters. (**a**) Simulation model of plasma device. (**b**) Voltage pulse applied to the powered electrode in this simulation.

**Fig. 4:**
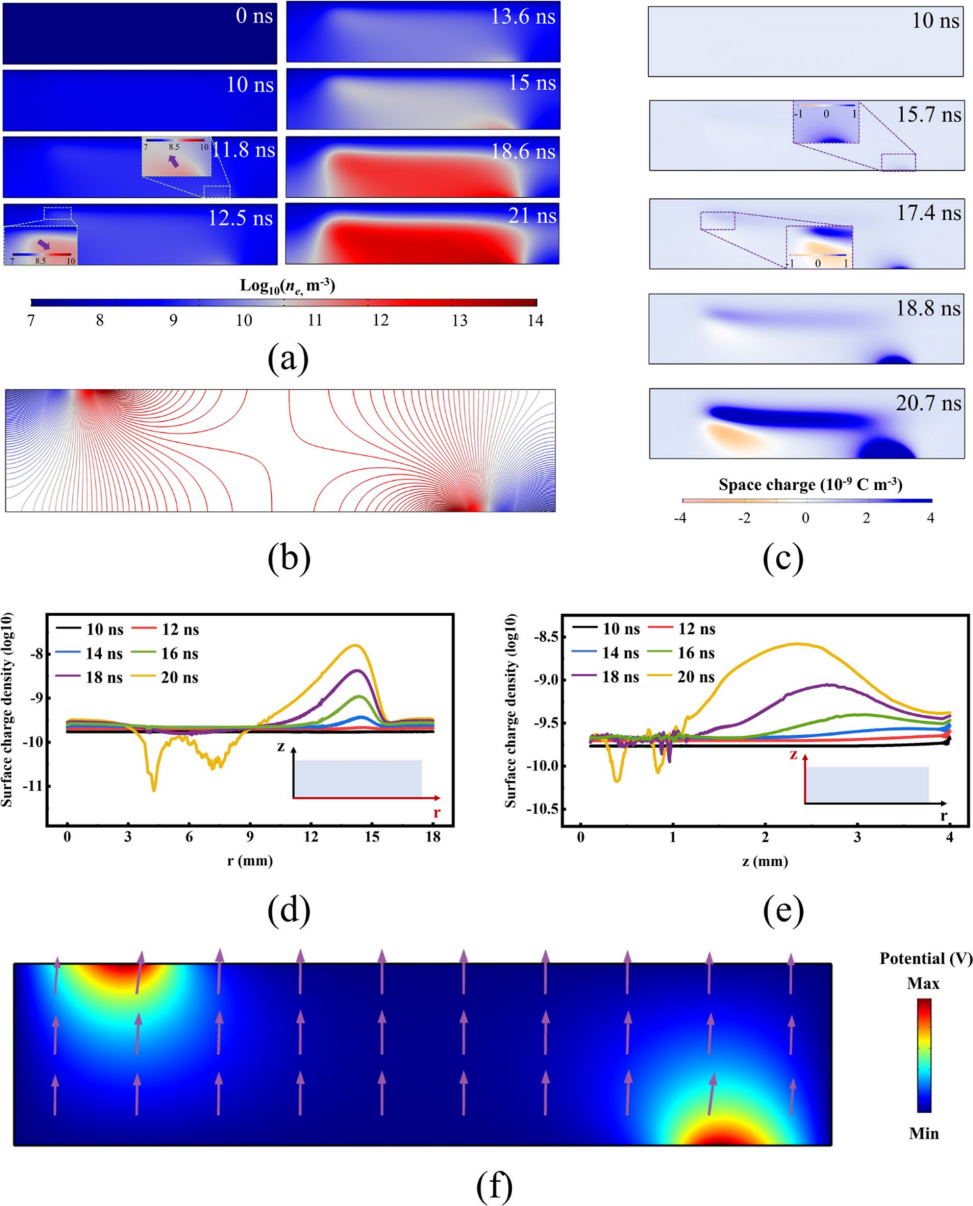
The evolution process of discharging. (**a**) Spatial distribution of log10 (n_e_) with n_e_ in m^− 3^. (**b**) Reduced electric field contour line. (**c**) Space charge in 10^− 9^ C m^-3^. Surface charge density at different times during a voltage pulse on (**d**) Radially, z = 1.1 mm, and (**e**) Axially, *r* = 9 mm. (**f**) Electric field strength and direction.

We established a two-dimensional fluid model to qualitatively analyze the discharge process of the device and the spatiotemporal evolution of active particles. The schematic of the model is shown in Fig. 3a, with the boundary conditions presented in Table 2. The applied voltage in this work is depicted in Fig. 3b. As shown in Fig. 4a, before a voltage was applied to the two electrodes (0 ns), the initial electron density within the discharge area was uniform (10^7^). As the voltage increased, weak ionization occurred in the air between the two electrodes. During this phase, although no discharge occurred, the density of seed electrons in the space increased due to pre-ionization (~ 5 × 10^8^). Once the applied voltage stabilized, at 11.8 ns, streamer formed near the high-voltage electrode. At a smaller scale (log10, 7 ~ 10), it can be observed that the streamer almost adhered to the discharge medium on the side of the high-voltage electrode, while propagating towards the central area of the discharge domain. Since the ground electrode is located at the upper end of the discharge area, a region of higher electric field intensity forms on the side of the ground electrode within the discharge area (Fig. 4b). At 12.5 ns, also at a smaller scale, streamers formed on the side of the ground electrode. Unlike on the high-voltage side, the streamers on the ground side were some distance from the boundary of the discharge area, influenced by the sheath and the magnitude of the electric field. As shown in Fig. 4b, the electric field intensity in the axial central region between the two discharge electrodes is significantly higher than on the sides, leading to the development of discharge towards the central area. At 13.6 ns (Fig. 4a), the discharge from the high-voltage electrode side connected with the induced discharge on the ground electrode side. The potential gradient near the high-voltage electrode was significantly greater than in other areas. At 15 ns (Fig. 4a), the electron density near the high-voltage electrode was higher than in other areas. Simultaneously, due to the high field strength gradient between the discharge electrodes, the streamers primarily propagated and intensified within the central area. In subsequent stages of voltage stabilization and reduction, the discharge continued to intensify within the discharge domain between the two electrodes.

The formation of high field strength areas led to the accumulation of space charge, with positive ions in the space moving directionally under the influence of the electric field, promoting the development of discharge. At 10 ns (Fig. 4c), although the streamers were not yet ignited, the gas within the discharge domain was weakly ionized due to the voltage applied to the electrodes. As the discharge occurred, this ionization became more intense. At 15.7 ns, a significant accumulation of positive charge occurred on the side of the positive electrode. At 17.4 ns, an accumulation of negative charge appeared on the side near the ground electrode. Generally, electrons are much less massive than ions (such as positive ions), hence, their migration and diffusion speeds are much faster than those of ions. As mentioned before, an induced discharge occurs near the ground electrode throughout the discharge process. When the induced discharge begins, electrons quickly move towards the anode, while the heavier positive ions move relatively slowly, leading to an accumulation of electrons near the ground electrode, creating a negative charge area. Due to intense electron collision reactions in plasma discharge, new positive ions are continuously produced in the discharge area, and some of the migrating and diffusing electrons are consumed by positive ions. This situation also leads to the development rate of the positive space charge accumulation area being greater than that of the negative space charge accumulation area (Figs. 4c and 18.8 ns). Theoretically, the direction of the electric field within the discharge area is generally from the ground electrode towards the high-voltage electrode, which may be the direction of development for the negative space charge accumulation area. In practice, due to the consumption of electrons by positive ions at the edges, the negative charge accumulation area experiences a slight shift (Figs. 4c and 20.7 ns).

The distribution of positive surface charge density, both radially (z = 1.1 mm, Fig. 4d) and axially (*r* = 9 mm, Fig. 4e), better describes the development trends of charge in the horizontal and vertical components. Radially, when the voltage reached a stable phase, more positive charges were produced near the high-voltage electrode (*r* = 15 mm) due to weak ionization of the gas compared to other areas. As the discharge progressed, the position of maximum positive charge accumulation continuously shifted to the left, and the peak increased, indicating radial development of the discharge. Within the interval of r approximately 3–9 (20 ns), there was a fluctuation in the distribution of positive charge density. This could be due to a substantial accumulation of negative charges in this interval, where electron drift and diffusion affected the distribution of positive charges. Similar to radial changes, the peak position of the axial distribution of positive charges also shifted over time. The difference is that the degree of axial shift seems to be much greater than that of the radial peak shift. This may be due to the vertical component of the electric field strength being much greater than the horizontal component throughout the discharge domain (Fig. 4f).

**Fig. 5:**
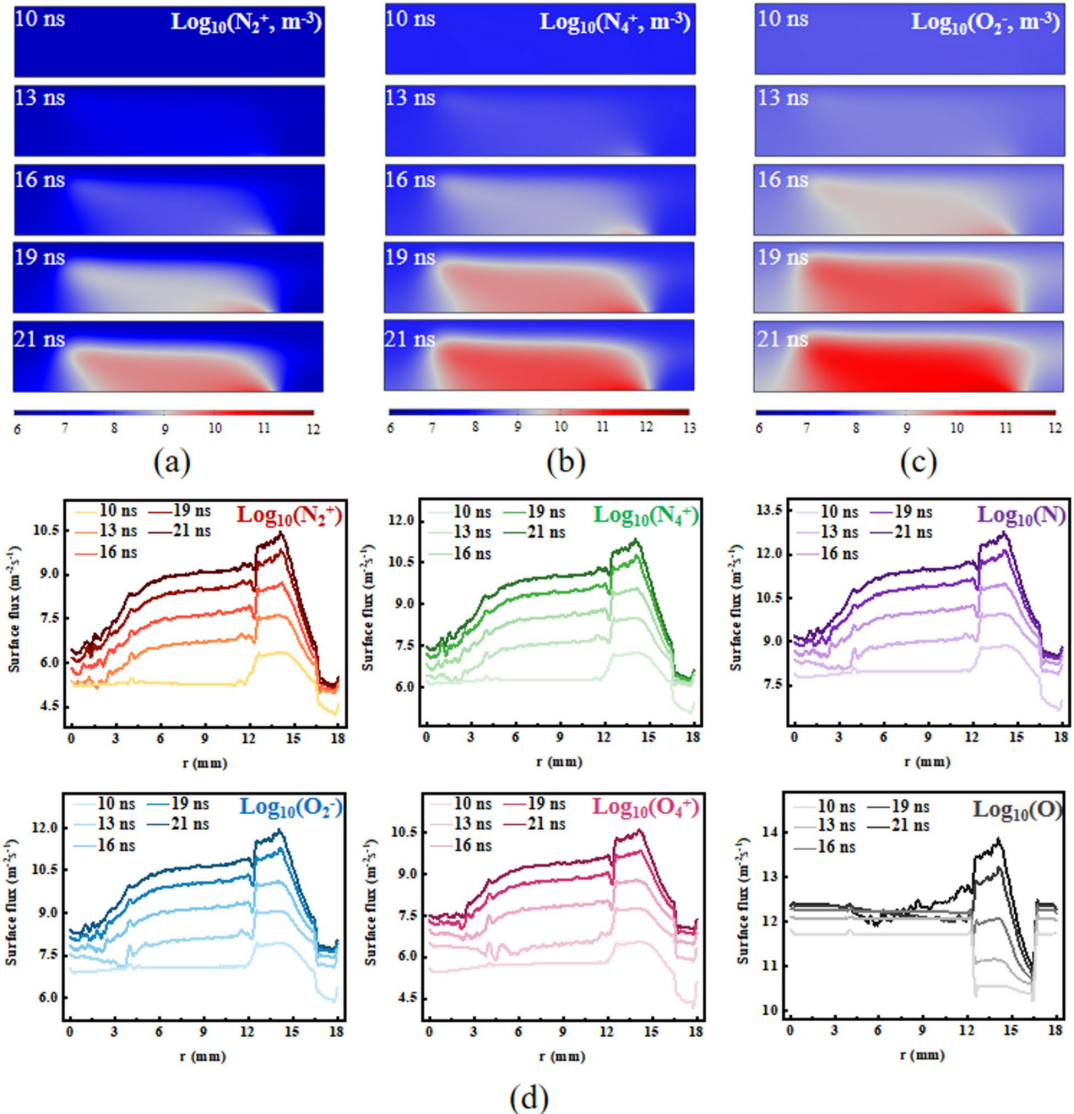
Spatial distribution of the number density (log10 scale) of (**a**) N_2_^+^, (**b**) N_4_^+^, and (**c**) O_2_^-^. (**d**) Temporal distribution of the surface flux of the reactive particles.

In the application of plasma biomedical technology, ROS and RNS play a pivotal role as active substances. However, these reactive particles generally have a short lifespan, making it challenging to detect them using experimental diagnostic methods. Therefore, we employed numerical simulations to elucidate the distribution patterns of these active particles. Due to the high ionization threshold, in plasma reactions, N_2_^+^ is typically produced by direct electron collision ionization of N_2_. Therefore, N_2_^+^ has the highest ion density in the most intense discharge areas, and the surface flux of N_2_^+^ also reflects this trend over time (Fig. 5a and d). N_4_^+^ is an extremely important positive ion, widely used in ion beam cancer therapy, biomedical material development, and drug delivery. From the distribution maps of ion density over time, N_4_^+^ exhibits a higher density and greater uniformity than N_2_^+^(Fig. 5b and d). This is because the main pathways for the formation of N_4_^+^ are the recombination and dimerization reactions of N_2_^+^ ions. Since electron collision ionization is not a pathway for generating N_4_^+^, the density of N_4_^+^ does not peak at the front of the streamers. This is also why the uniformity of N_4_^+^ ion density is far superior to that of N_2_^+^ ions. In addition to dimerization reactions, Penning ionization between excited states of N_2_ is also an important production pathway for N_4_^+^. Unlike other reactions, the rate of this reaction depends not on the electric field strength but on the density of N_2_. As an electronegative gas, O_2_ consumes electrons through adsorption reactions to produce O_2_^-^ and O^-^. A fairly uniform spatial distribution of the produced O_2_^-^ ions was formed (Fig. 5c and d).

### Degradation effect

**Fig. 6:**
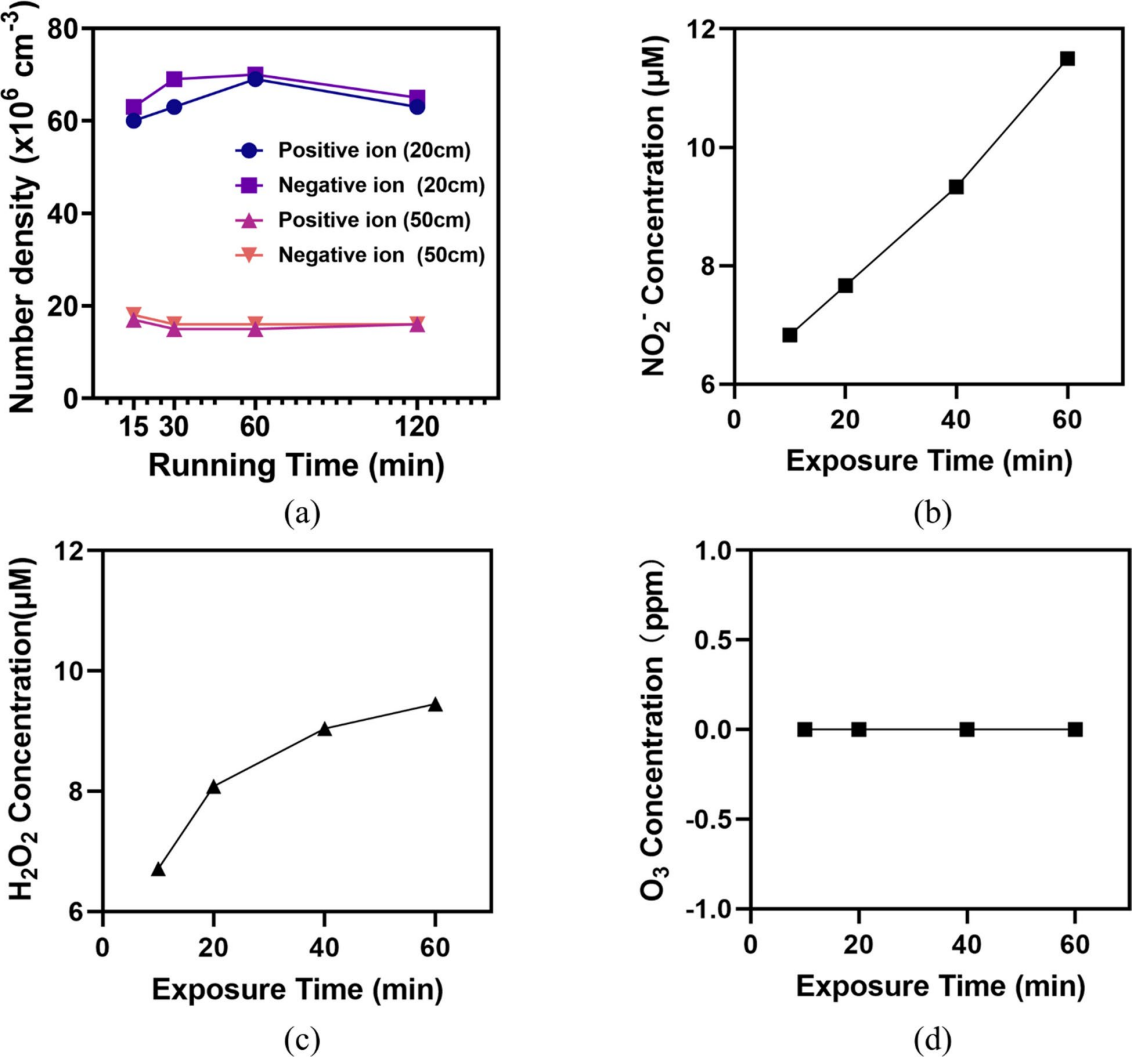
(**a**) The concentration of positive and negative ions in the air measured at two different distances to the plasma outlet. (**b**) Concentration of NO_2_^-^ in water. (**c**) Concentration of H_2_O_2_ in water. (**d**) Concentration of O_3_ in the air.

**Fig. 7:**
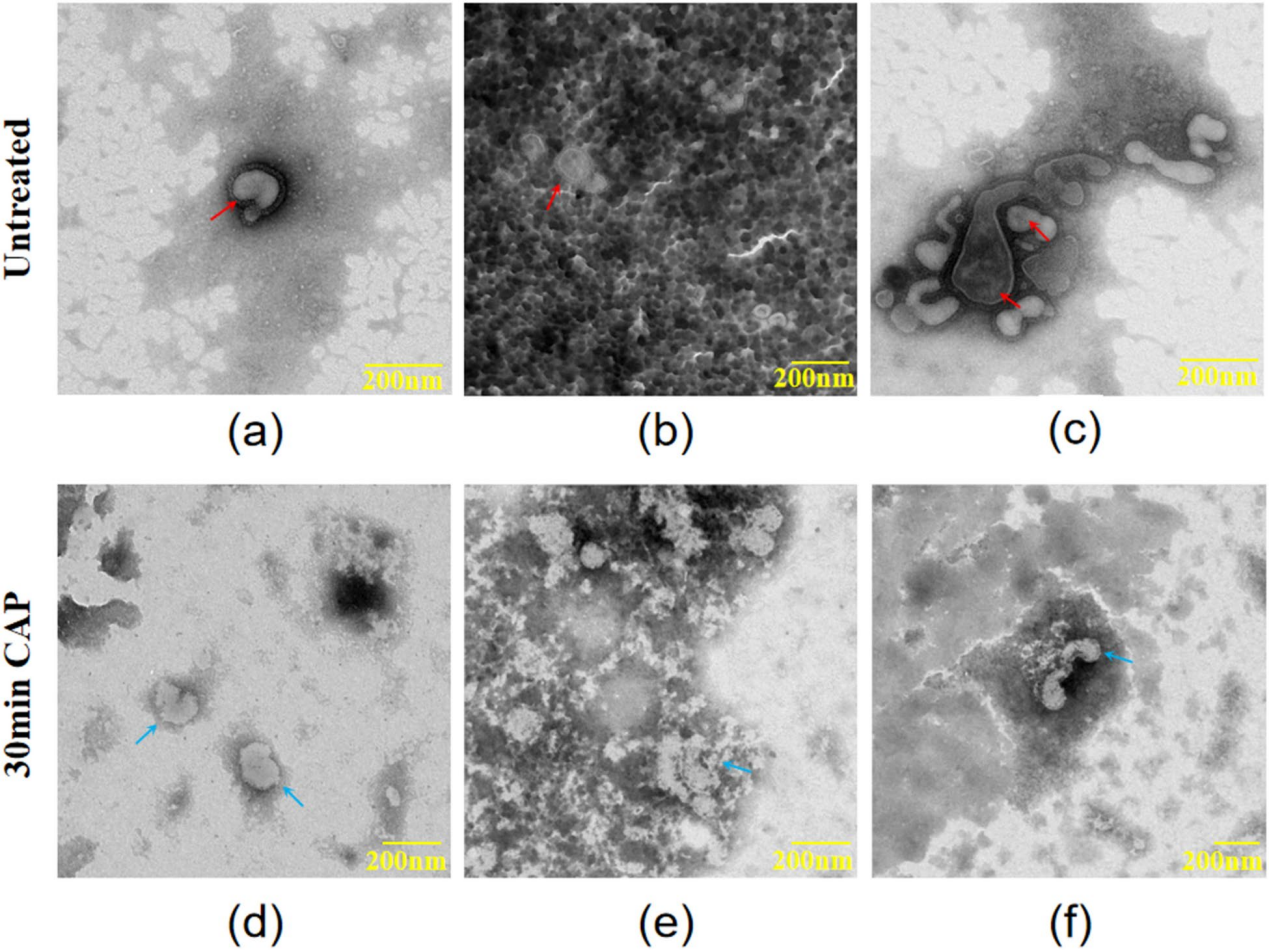
TEM images of SARS-CoV-2 virus before and after plasma treatment. (**a**) Spikes are clearly seen. (**b**) The virus are distributed in the medium. (**c**) Mixed and multi-shaped virion groups with bright and dark vision areas. (**d**) Protein edges become clear and denaturized with spikes morphology disappear. (**e**) The coronavirus bodies are indistinguishable from the background. (**f**) The capsid protein of groups of coronaviruses has been denatured and clustered with no dark or bright areas.

By monitoring the concentration of total positive and negative ions over time through an air ion counter, it was found that the device reaches a stable state after 30 min of continuous operation (Fig. 6a). Within 60 min of exposure, the concentration of long-lived species NO_2_^-^, and H_2_O_2_ were found be to around 10 μm, measured in 2 mL of water with 20 cm of distance to the air outlet (Fig. 6b and c). O_3_ in gas-phase was at least not detectable by an ozone meter in the air (Fig. 6d) in this experiment. Figure 7a displays a TEM image of SARS-CoV-2 with spikes, the diameter is approximately 100 nm. SARS-CoV-2 virion has a scattered dispersion in the culture medium, and the clear and pervasive spikes can be seen and differentiated from the background medium (Fig. 7b, **Supplementary Fig. ****S1**). As shown in Fig. 7c and **Supplementary Fig. S2**, there are mixed shapes of coronavirus clusters, such as spherical and hook-shaped, exhibiting light and dark virions. After 30 min of plasma treatment, the typical spikes of SARS-CoV-2 disappeared, and the edge proteins of SARS-CoV-2 viral body were clear (Fig. 7d). It was hard to distinguish the virus from the background medium anymore (Fig. 7e**)**, and groups of protein capsids have been found to be denatured, and there were no distinct regions of bright and dark of SARS-CoV-2 (Fig. 7f, **Supplementary Fig. S3**). At the same time, it is even difficult to distinguish the protein body of the coronavirus from the background in some areas. Multiple coronaviruses were condensed together after being denatured by plasma treatment. Therefore, the coronavirus protein has undergone irreversible modification after plasma treatment, and thus killed by plasma treatment.

### Safety assessment

Rats were divided into four groups, with 0, 2, 3, and 4 weeks of plasma exposure, respectively. Hematological examination was conducted for blood biochemical and routine examination (Table 3 and **Supplementary Table S2**). It can be seen from Table 3 that most of the values of biochemical blood indices in the experimental groups were lower than those in the control group. The serum creatinine (CR) in 2-week and 3-week groups, and high-density lipoprotein cholesterol (HDL-C) in 2-week group, significantly decreased compared with those in the control group (*p* < 0.01). CR in 4-week, total protein (TP) in 2-week, HDL-C in 4-week, aspartate aminotransferase (AST) in 2-week and 4-week, as well as alanine aminotransferase (ALT) in 2-week also presented significant reduction (*p* < 0.05). The results show that after 4 weeks of exposure to plasma, levels of CR, HDL-C, and AST in rats were significantly down-regulated, however, still within the normal range. Theoretically, slight reduction of CR usually does not have typical clinical significance. While simultaneous reduction of the three may be related to malnutrition due to the 16 h of fast. The situation, however, alleviated in the 4-week group compared with the 2-week group, indicating potentially eased adaptive stress after 4-week of plasma exposure. Other indicators, including glucose (GLU), blood urea nitrogen (BUN), uric acid (UA), low density lipoprotein-cholesterol (LDL-C), and albumin (ALB), were found not changed significantly.

**Table 3:** Biochemical blood indicators after 0 (control), 2, 3 and 4 weeks of plasma exposure.

Parameters	0 week	2 weeks	3 weeks	4 weeks
CR(µmol L^− 1^)	73.9 ± 8.46	49.88 ± 4.53^**^	51.72 ± 3.76^**^	57.13 ± 1.96^*^
GLU(mmol L^− 1^)	8.19 ± 2.29	5.76 ± 0.56	6.97 ± 1.22	7.95 ± 1.84
BUN(mmol L^− 1^)	7.19 ± 1.24	5.61 ± 0.81	5.93 ± 1.19	7.75 ± 1.33
TP(g L^− 1^)	57.56 ± 3.23	52.4 ± 2.84^*^	54.72 ± 2.27	55.35 ± 0.57
UA(µmol L^− 1^)	260.43 ± 15	243.4 ± 42.67	270.92 ± 29.53	269.43 ± 23.27
HDL-C(mmol L^− 1^)	0.83 ± 0.05	0.58 ± 0.1^**^	0.78 ± 0.14	0.72 ± 0.07^*^
LDL-C(mmol L^− 1^)	0.33 ± 0.05	0.27 ± 0.08	0.43 ± 0.11	0.32 ± 0.03
AST(U L^− 1^)	212.87 ± 34.58	142.96 ± 27.69^*^	167.54 ± 46.37	149.75 ± 14.26^*^
ALT(U L^− 1^)	53.33 ± 12.02	25.62 ± 7.26^*^	44.26 ± 14.42	40.85 ± 2.42
ALB(g L^− 1^)	25.16 ± 1.92	24.82 ± 1.25	25 ± 1.24	24.23 ± 0.29

* *p* < 0.05; ** *p* < 0.01; t-test, *n* = 5 (3 females and 2 males) in each group.

**Fig. 8:**
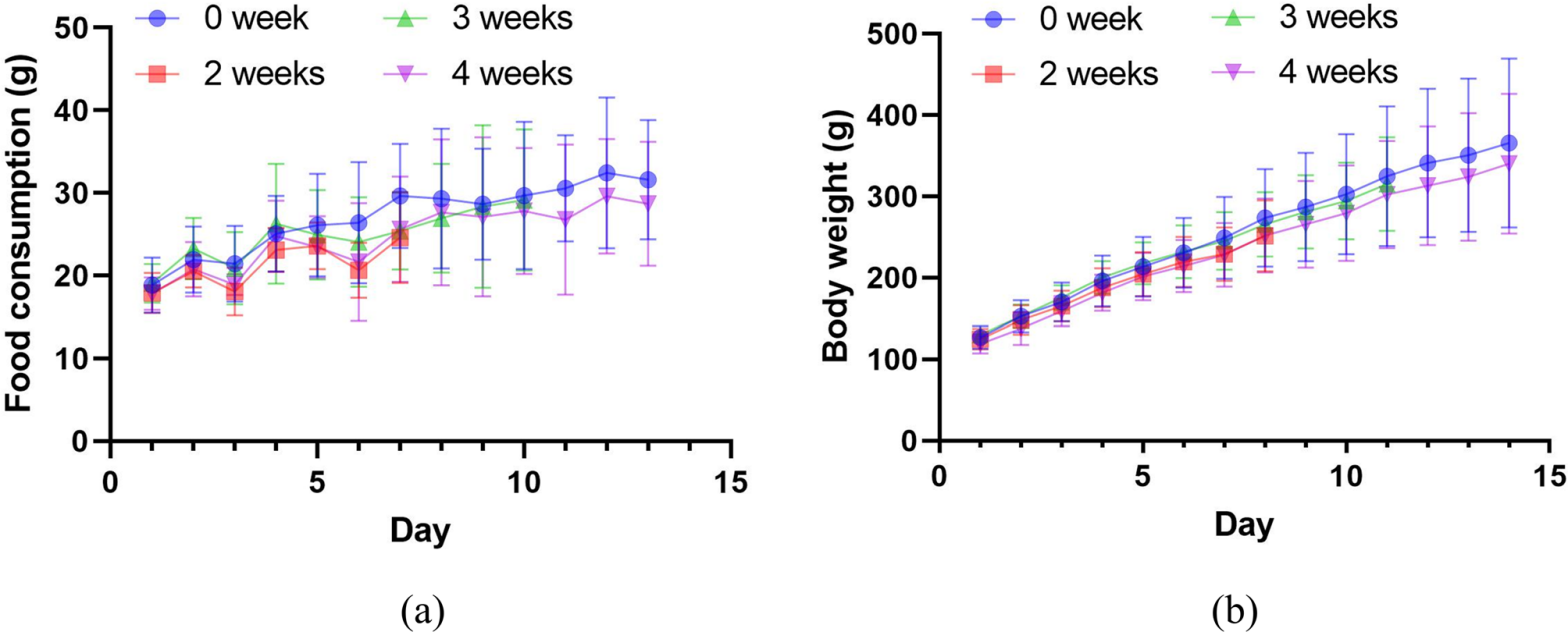
Follow-up of rats. (**a**) Food consumption changes. (**b**) Body weight change.

Food consumption of rats is shown in Fig. 8a. The test process followed the SPF level barrier system laboratory with the license (SYXK (Zhe) 2019-0011). Rats were fed with Co60 sterilized nutrient compound feed and water, and illuminated at intervals of 12 h. From Fig. 8b, the control group and 4-week group have a similar trend of average body weight increasing as well as in both male and female groups (**Supplementary Fig. S4**). Breathing plasma and enduring plasma irradiation for a long time have almost no effect on rats’ food consumption. Overall, a plasma-fulfilling environment, including ROS, RNS, and other ions, didn’t influence rats on both food consumption and body weight.

**Fig. 9:**
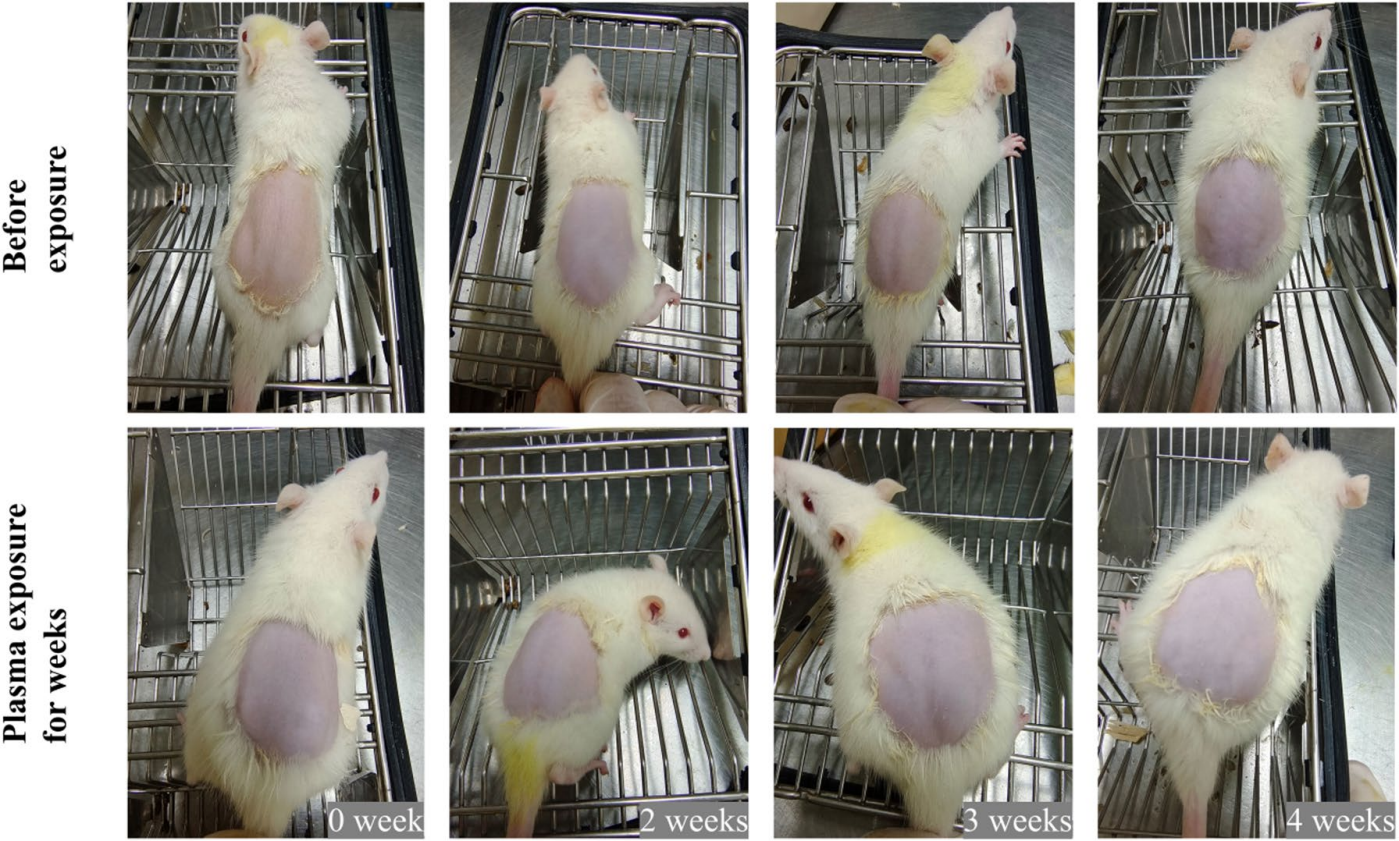
Comparison of skin changes before and after weeks of plasma exposure.

**Fig. 10:**
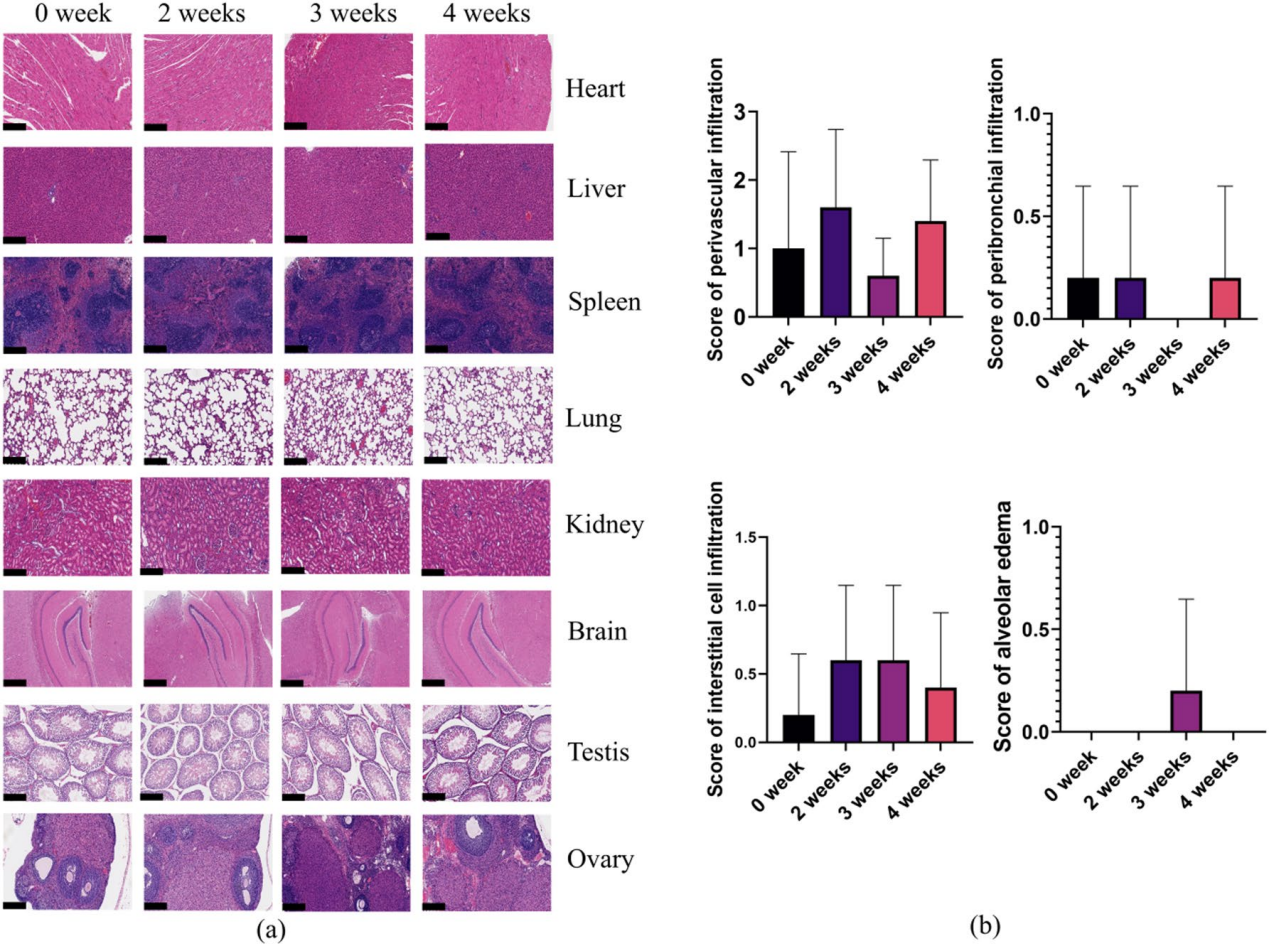
(**a**) HE staining images of major organs. The images of the brain are in 40×, while the rest of the HE staining images are in 100×. (**b**) Comparison of the degree of pathological changes in lung in each group of rats.

Rats after plasma treatment for 4 weeks, their mental and behavioral performances were normal. There was no diarrhea, hair loss, or death. Figure 9 shows no changes on the back skin of the rats, such as skin dryness, aging, and telangiectasia, after 4 weeks of plasma exposure. Figure 10a shows HE staining of major organs, including male rats’ testis and female rats’ ovary, before and after weeks of plasma exposure. No pathological effects on male rats’ testis, female rats’ ovary were found, nor in the heart, lung, stomach, liver, and brain of rats. Particularly, airway inflammation, perivascular / peribronchial infiltration, interstitial cell infiltration, and alveolar edema in the lung tissue were conducted blinded scoring, with airway inflammation in all slices scored 0, and others shown no significant difference compared with the 0-week group (Fig. 10b**).** Potential pathological features in other body systems were investigated as well, and no signs presented (**Supplementary Fig. S5** - **Fig. S12**).

## Discussion

CAP has received considerable attention for its potential biomedical applications. Emerging application fields of CAP include, but not limited to, wound healing, sterilization of infected tissue, inactivation of microorganisms, tooth bleaching, blood coagulation, skin regeneration, neural protection, and cancer therapy^25–33^. Plasma contains the energized ions, free radicals, reactive species, UV radiation, and the transient electric fields inherent with plasma delivery, which interact with the cells and other living organisms^34–38^. Fig. 2a indicates that UV photons are not the major plasma species with our setup. Major plasma-generated reactive species include superoxide (O_2_^-^), nitric oxide (NO), atomic oxygen (O), ozone (O_3_), hydroxyl radical (•OH), singlet delta oxygen (SOD, O_2_(^1^∆g)), peroxynitrite (ONOO^-^), hydrogen peroxide (H_2_O_2_), nitrite (NO_2_^-^), etc^39–43^. SARS-CoV-2 infection relies on recognition of and binding to the cellular receptor hACE2 through receptor binding domain (RBD) of the spike protein, and disruption of this process can effectively inhibit SARS-CoV-2 invasion^44^. Guo et al. applied plasma-activated water to inhibit pseudo virus infection through S protein inactivation^24^. Qin et al. identified plasma-generated reactive species inactivated the RBD of SARS-CoV-2 spike protein, which is responsible for the recognition and binding to hACE2^45^. While our results indicated that the typical spikes of SARS-CoV-2 disappeared and the edge of the protein body of SARS-CoV-2 got clear after plasma treatment (Fig. 7). The coronavirus proteins not only spike protein, undergone irreversible modification after plasma treatment.

Given that SARS-CoV-2 virus have an liquid-based interior environment, and plasma components mostly transfer to long-lived species such as NO_2_^-^, NO_3_^-^, and H_2_O_2_ in water conditions. The concentration of NO_2_^-^, and H_2_O_2_, which have been demonstrated to have virus killing effect, have been measured in water with 20 cm of distance to the plasma outlet. O_3_, another potential effector for killing virus but at the same time induce biological safety issues was also measured in gas-phase by an ozone meter in the air with 0.0 accuracy. The results show that there’s no detectable O_3_, indicating that other RONS were the major components for disinfection of the SARS-CoV-2 virus. Excited nitrogen in the OES explains the origin of NO_2_^-^, and OH recognized from OES suggests the origin of H_2_O_2_. Qin et al.^19^ showed effectiveness of O_3_ and H_2_O_2_ in damaging spike protein of pseudotyped SARS-CoV-2 variants and coronavirus GX_P2V in waste water. Reema et al.^46^ also give proof that H_2_O_2_ play more roles in deactivation of spike protein and RBD proteins of Omicron, leading to less binding with ACE2 receptor. In comparison, Guo et al.^24^ demonstrated that ozone water exhibited little inactivation effect on RBD, while short-lived ONOO- played crucial roles in inactivation of SARS-CoV-2 spike proteins, thus inhibiting its binding to hACE2. The limited availability of detection probes for short-lived reactive species currently hinders the advancement of full knowledge in this field and worth further efforts.

Our observations also indicated that rats undergoing plasma exposure and breathing lots of reactive species do not develop runny noses, itchy eyes, and scratchy throats. In addition, their mental and behavioral performances were as normal as usual, and rats had no diarrhea, hair loss, or death. The plasma does not affect the rats’ daily physiological behavior, body weight, food consumption, and organ histopathology (Fig. 8**and** Fig. 10). When rats breathe in, reactive species move through the nose or mouth, down the throat into the trachea, and then into the lungs^47^. The pathological section of the bronchi did not show the muscles around the bronchi tighten or the lining of the bronchi swell. Reactive species come into the lungs easily and pass directly from the alveoli in the lungs into the bloodstream. The biochemical blood indicators showed no major change after breathing reactive species for a long time (Table 3), although with statistically significant reduction in CR, HDL-C and AST with as long as 4-week of exposure. Given that no difference were observed in food consuming and body weight, the effect of all indicators in the normal range and the rose back of the values compared with 2-week exposure, the reduction may most likely to be a combined result of fast before sample collection and stress response, those need further investigation. In addition, previous studies also have supported that direct plasma treatment of blood induces blood coagulation, whereas breathing plasma almost did not induce changes in biochemical blood indicators^48,49^. Plasma did not cause changes in the system that control the heart beats, and it did not induce plaque which break off the wall of the blood vessel and block blood flow. Plasma caused no changes on the back skin of the rats, such as skin dryness, aging, and telangiectasia (Fig. 9). Breathing plasma for a long time and enduring plasma irradiation for a long time have no pathological effect on the testis, ovary, heart, lung, stomach, liver, and brain (Fig. 10). In addition, plasma did not cause inflammation throughout the body, nor decrease blood flow, loosen plaque, or trigger a blood clot. Headaches, anxiety, or harms to the central nervous system have not been found.

The operation of air-fed nonthermal plasmas yields an array of oxidants, including ozone (O_3_), NO / NO_2_, and peroxides. While these species are relevant for neutralizing viruses, they are also responsible for potentially deleterious health effects upon inhalation. We have performed ozone measurement by using an ozone meter in the air with 0.0 accuracy (Fig. 6d). The meter was placed near the air outlet of the device, and no detectable O_3_ was found with this meter resolution. The experimenter also reported that almost no toxic smell when the device was in charge. However, this doesn’t mean there is no ozone at all for this device. It’s hard to predict the result if an ozone meter with finer resolution was used, and if the measurement was conducted in an small and closed environment. For NO and peroxides, concentrations of NO_2_^-^, and H_2_O_2_ were measured in device-treated water. As NO has very short lifetime (ns) and is very unstable, they finally generate NO_2_^-^ after metabolism in the human body. The concentrations of NO_2_^-^ thus often a representative of that of NO. The results showed increased NO concentration and gradually saturated H_2_O_2_ concentration within 60 min of exposure. NO_2_ by theory can be generated in high-voltage discharge through reaction of NO and O_2_. However, we did not see the NO peaks (200–300 nm) in the OES spectrum (Fig. 2a). This indicates that there may not be too much NO_2_ generated in the air, and the NO_2_^-^ we detected may mostly are the reaction products of other RNS in water. For H_2_O_2_, as we put water under the air outlet, the change of environment humidity may exert an amplification effect and thus more H_2_O_2_ can be generated and detected. The indoor exposure limits are 0.00016 ppm for O_3_, 0.0002 ppm for NO_2_ within one hour average according to the newest version China’s indoor air quality standard GB/T 18,883 − 2022. According to the standard, safety of continuous human exposure to this device need to be investigated and more data should be given in later research.

## Conclusions

In conclusion, TEM results show that the typical spikes of the processed coronaviruses have disappeared, and the edge proteins of SARS-CoV-2 body were clear after plasma treatment. The coronavirus proteins, not only spike proteins, undergo irreversible denaturation by plasma treatment. By analyzing the emission spectra of the discharge and performing numerical simulations, it was demonstrated that the plasma generator produces significant amounts of ROS/RNS, along with positive and negative ions that play a dominant role in viral inactivation. The discharge between the two electrodes typically initiates at the high-voltage electrode and propagates towards the grounded electrode, ultimately forming a relatively uniform region of high electron density between the electrodes. Breathing reactive species generated by plasma for a long time and enduring plasma irradiation for a long time do not affect the rats’ daily physiological behavior, body weight, food consumption, and organ histopathology. The level of CR, HDL-C and AST lowered which worth further investigation, while other indicators have no significant changes. In all, the CAP device with air-feeding gas that we developed can be effectively used for SARS-CoV-2 virus inactivation without obvious acute toxicity in rats.

## Methods

### Device characterization

The discharge voltage and current were monitored by a Tektronix P6015A high-voltage probe and a Tektronix A621 current probe, respectively, and recorded by a Tektronix TPS2024B digital oscilloscope. Imaging of the device discharge was performed in the absence of ambient light using a Canon 600D digital camera. For OES measurement, the plasma generator was placed in air and subjected to discharge, with spectral sampling conducted at the corona region near the electrode tip. Spectral data in the range of 250 nm to 900 nm were collected by using a spectrometer (BIM-6602 A-02, Brolight) equipped with a linear CMOS camera Hamamatsu S11639. A fiber (SIM-6102-1020-S/T-P) was used to focus the plasma light into the spectrometer. For the H_β_ fine spectrum, the plasma generator is placed within a vacuum chamber, where hydrogen gas is introduced before initiating the discharge. The hydrogen discharge plasma emission spectra are collected in a dark environment to shield against external stray light. The light is focused onto the fiber optic head through a lens assembly with 55 mm focal length, and the spectral data in the range of 450–700 nm is detected using a high-sensitivity PMT (Photomultiplier Tube) monochromator (44 W, Teledyne Princeton Instruments). Calibration was performed using an LHM254 mercury lamp source prior to detection. Thermal imaging was captured by an professional thermal imager UTi320E (UNI-T). The positive and negative ions were counted by an air ion counter Model AIC200M (Alphalab Inc.) And ozone was detected through an O_3_ gas detector ADKS-1 (AIKESI). Concentration of NO_2_^-^ and H_2_O_2_ in the water were detected by reagent S0021 and S0038 (Beyotime), respectively.

### Virus cultivation and inactivation

Vero E6 cells were cultivated in a humidified incubator under 37 °C with 5% CO_2_. DMEM medium containing fetal bovine serum (FBS) (10% ), penicillin (100 U mL^-1^ ) and streptomycin (Beyotime #C0222) (100 µg mL^-1^) was used. The titer of the original coronavirus (SARS-CoV-2, WIV04, GenBank: MN996528.1) was 1 × 10^7~8^ TCID_50_ mL^-1^, and the concentration of fetal bovine serum (FBS) in the virus stock solution was about 2%. The plasma fan was put into the safety cabinet. Six small dishes of 3 cm diameter were prepared with a piece of gauze (2 cm×2 cm in size, sterilized and dried) in the center of each dish. We added disease venom (10 µl) to the center of the gauze, waited for the disease venom to disperse, and then fixed the gauze in the small dish. Four small dishes were put into the tray under the plasma fan, and the distance between the four small dishes and the plasma fan outlet was 20 cm. The plasma fan was turned on and timed. After 30 min of action, the dishes were removed for subsequent virus elution and testing. The rest two small dishes were placed in the safety cabinet for 60 min without the plasma fan operating, and then virus elution was carried out as a control.

### TEM imaging

After virus extraction, suspend the virus in PBS buffer. Following sample preparation, use TEM (Tecnai Spirit D1297) to observe at different magnifications.

### Experimental animals

This study was approved by the Institutional Animal Care and Use Committee (IACUC), ZJCLA with Application No. ZJCLA-IACUC-20,020,181. All experiments were carried out in accordance with the relevant guidelines and regulations, including the Animal Research: Reporting of In Vivo Experiments guidelines (ARRIVE 2.0).

Twenty SPF SD rats, including 12 females and 8 males, weighing 100 ~ 132 g, were provided by the Institutional Animal Care and Use Committee (IACUC), ZJCLA under Application No. ZJCLA-IACUC-20,020,181. They were in good health after adaptation to the animal house. Feeding conditions: The test process was completed in the SPF-grade barrier system laboratory, license number: SYXK(Zhejiang)2019-0011. The rats were fed the Co60 sterilized nutrient compound feed, drank water freely, and were illuminated at a 12 h interval. 20 SD rats were shaved with hair removal cream on the back with removal areas of more than 2 cm×3 cm. They were randomly divided into 4 groups with 5 rats in each group, including 3 females and 2 males, by using the completely random grouping method by a separate individual experimental operator. The experimental group was divided into 3 groups: 2-week irradiation group, 3-week irradiation group, 4-week irradiation group, and the control group marked as 0-week irradiation. Blood samples were collected post exposure after 16 h of fast. Anticoagulant whole blood was collected from the inner canthus vein of the eye. Major organs were collected and stained by HE staining. The lung tissues were blindly scored from 0 to 5 by an experimentalist who was unaware of the grouping situation. Of which Score 0 denotes the absence of observable lesions and tissue appears normal with no detectable pathological change. Score 1 indicates very small or barely detectable changes, and lesion affects only a few cells or a very limited area and is considered to have no biological significance. Score 2 represents a mild lesion that is limited in extent and/or severity, accompanied by slight alterations in tissue architecture and function. Score 3 corresponds to a distinct lesion involving a moderate portion of the tissue, with evident structural abnormalities and a clear impact on function. Score 4 signifies a marked and extensive or severe lesion, characterized by prominent tissue damage and significant functional impairment. Score 5: reflects a massive and diffuse lesion with severe tissue destruction and profound loss of function. Data from 20 rats were all presented and without exclusion. The data were statistically tested using SPSS11.5, and were analyzed by t-test between two groups and F-test among three or more groups, with one asterisk indicates *p* < 0.05 and two asterisks indicate *p* < 0.01.

### Plasma exposure

The plasma generator was previously fixed on the top of the rat’s stainless steel cage with the outlet facing the cage. All irradiated rats were placed in a bechtop (**Supplementary Fig. S13**). The plasma generator was started first, and after 15 min and within 1 h, the air ion counter was used to detect the density of positive and negative ions in the experiment cabin for several times, and the concentration of positive and negative ions were recorded after the detection was completed. The test animals were then placed in the pod. The control group was placed in the normal environment, and the experimental group was placed in the above environment according to the group time. The animals were fed in stainless steel cages and ate freely. The food intake and weight of each rat were recorded 3 times a week.

## Supplementary Information

Below is the link to the electronic supplementary material.

Supplementary Material 1

## Data Availability

Data are available from the corresponding author Dr Fei CAO (f.cao@siat.ac.cn) upon reasonable request.
