# Supplementary material for: Air-fed cold atmospheric plasma device as a safe and effective anti-SARS-CoV-2 air filter
Source: Sci Rep. 2026 Jan 12;16:5038. doi: 10.1038/s41598-026-36088-y (PMC12876883; doi:10.1038/s41598-026-36088-y)
Supplement: Supplementary file 1 — Supplementary Material 1 [file 41598_2026_36088_MOESM1_ESM.docx]

Supporting Information

Air-fed cold atmospheric plasma device as a safe and effective anti-SARS-CoV-2 air filter

Fei Cao#*, An Yan#, Qingnan Xu, Qiujie Fang, Xiao Chen, Shuang Xue, Longfei Qie, Jie Fang, Gbenga A. Martins, Ye Lu, Jamoliddin Razzokov, Ruixue Wang, Guiqiang Wang*, and Zhitong Chen

**Table S1.** Assessment of device charge leakage.


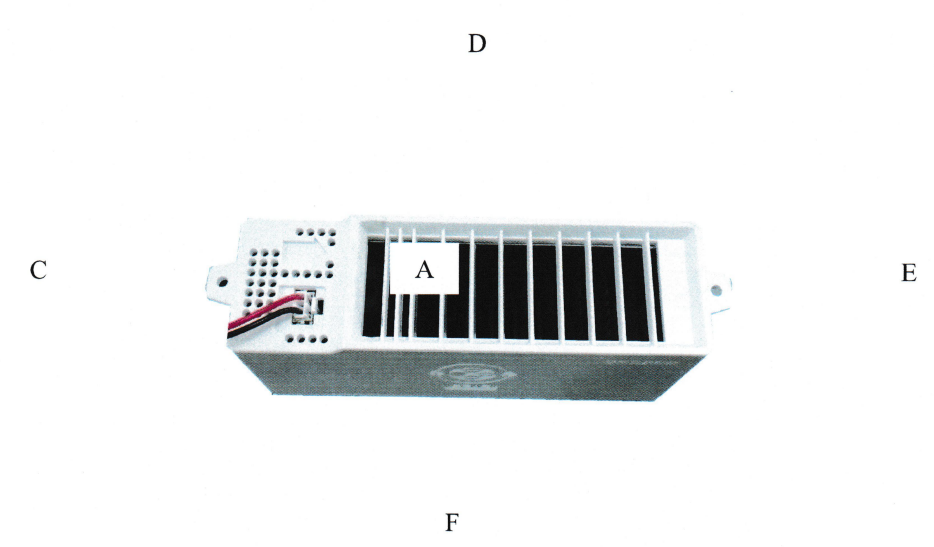


| Measured points | A | B | C | D | E | F | G | H |
| --- | --- | --- | --- | --- | --- | --- | --- | --- |
| Charge leakage index | 0.28 | 0.26 | 0.22 | 0.21 | 0.23 | 0.22 | 0.21 | 0.20 |

**Table S2.** Blood routine examination.

|  | WBC | RBC | HGB | MCV | PLT | NEU% | LYM% | MON% | EOS% | BAS% | NEU# | LYM# | MON# | EOS# | BAS# | HCT  % | MCH | MCHC | RDW-CV % | RDW-SD | MPV | PCT % | PDW | P-LCR | P-LCC |
| --- | --- | --- | --- | --- | --- | --- | --- | --- | --- | --- | --- | --- | --- | --- | --- | --- | --- | --- | --- | --- | --- | --- | --- | --- | --- |
| 4-week-5 | 3.39 | 6.55 | 151 | 55.9 | 719 | 18.7 | 56 | 24.3 | 0.7 | 0.3 | 0.64 | 1.9↓ | 0.82 | 0.02 | 0.01 | 36.6 | 23.1 | 413 | 8 | 29 | 9.2↑ | 0.661 | 10.6 | 0.178 | 128↑ |
| 4-week-4 | 5.95 | 5.92 | 139 | 50.1 | 490 | 14.3 | 69.2 | 15.3 | 0.6 | 0.6 | 0.84 | 4.12 | 0.91 | 0.04 | 0.04 | 29.7↓ | 23.5 | 468↑ | 7.9 | 27.1 | 9.1 | 0.446 | 9.9 | 0.157 | 77 |
| 4-week-3 | 5.43 | 6.33 | 138 | 51.4 | 667 | 12.1 | 75.9 | 10.7 | 0.8 | 0.5 | 0.66 | 4.12 | 0.58 | 0.04 | 0.03 | 32.5↓ | 21.8 | 425 | 7.9 | 27.1 | 8.8 | 0.587 | 10.6 | 0.162 | 108↑ |
| 4-week-2 | 10.58 | 6.38 | 151 | 52.2 | 777 | 10.5 | 77 | 11.6 | 0.5 | 0.4 | 1.11 | 8.15 | 1.23 | 0.05 | 0.04 | 33.3↓ | 23.7 | 453 | 7.9 | 27.1 | 8.9 | 0.692 | 10.3 | 0.16 | 124↑ |
| 4-week-1 | 7.33 | 6.18 | 147 | 52.3 | 660 | 10.8 | 78.2 | 10.1 | 0.4↓ | 0.5 | 0.79 | 5.73 | 0.74 | 0.03 | 0.04 | 32.3↓ | 23.8 | 455 | 8.4 | 27.1 | 8.7 | 0.574 | 9.8 | 0.141 | 93 |
| 0-week-5 | 4.68 | 7.6 | 173 | 50.9 | 600 | 23.9 | 53 | 22 | 0.6 | 0.5 | 1.12 | 2.48↓ | 1.03 | 0.03 | 0.02 | 38.7 | 22.8 | 447 | 7 | 27.1 | 9.8↑ | 0.588 | 12.6 | 0.241 | 145↑ |
| 0-week-4 | 2.91 | 6.55 | 158 | 55 | 746 | 20.6 | 53.7 | 24.2 | 1 | 0.5 | 0.61 | 1.56↓ | 0.7 | 0.03 | 0.01 | 36 | 24.1 | 439 | 7.5 | 29 | 9.1 | 0.679 | 10.1 | 0.152 | 113↑ |
| 0-week-3 | 3.39 | 6.76 | 155 | 51.2 | 678 | 16.4 | 61.1 | 21.1 | 0.7 | 0.7 | 0.56 | 2.07↓ | 0.72 | 0.02 | 0.02 | 34.6↓ | 22.9 | 448 | 7.7 | 27.1 | 8.6 | 0.583 | 10.1 | 0.144 | 98 |
| 0-week-2 | 4.42 | 6.35 | 155 | 54.5 | 709 | 18.8 | 65.5 | 14.6 | 0.7 | 0.4 | 0.82 | 2.9 | 0.65 | 0.03 | 0.02 | 34.6↓ | 24.4 | 448 | 8 | 29 | 9.2↑ | 0.652 | 10.6 | 0.175 | 124↑ |
| 0-week-1 | 3.44 | 6.52 | 148 | 49.8 | 633 | 30.8 | 46.3 | 21.2 | 1.2↑ | 0.5 | 1.06 | 1.59↓ | 0.73 | 0.04 | 0.02 | 32.5↓ | 22.7 | 455 | 8.7 | 27.1 | 9.5↑ | 0.601 | 11.1 | 0.193 | 122↑ |
| 3-week-5 | 2.89↓ | 6.45 | 135 | 54.3 | 766 | 19.2 | 62.3 | 17.3 | 0.8 | 0.4 | 0.56 | 1.8↓ | 0.5 | 0.02 | 0.01 | 35↓ | 20.9 | 386 | 8.2 | 29 | 9.1 | 0.697 | 10.4 | 0.169 | 129↑ |
| 3-week-4 | 3.69 | 6.57 | 138 | 54.9 | 764 | 14 | 69.9 | 14.9 | 0.9 | 0.3 | 0.52 | 2.58↓ | 0.55 | 0.03 | 0.01 | 36.1 | 21 | 382 | 8 | 29.6 | 8.7 | 0.665 | 10.3 | 0.151 | 115↑ |
| 3-week-3 | 3.04 | 6.18 | 135 | 56.4 | 650 | 18 | 62.4 | 17.9 | 1.3↑ | 0.4 | 0.55 | 1.9↓ | 0.54 | 0.04 | 0.01 | 34.9↓ | 21.8 | 387 | 7.1 | 29.6 | 9.4↑ | 0.611 | 10.9 | 0.188 | 122↑ |
| 3-week-2 | 9.22 | 5.38 | 130 | 63.3 | 1152 | 9.4 | 70.8 | 19.3 | 0.2↓ | 0.3 | 0.86 | 6.53 | 1.78↑ | 0.02 | 0.03 | 34.1↓ | 24.2 | 381 | 12.8 | 32 | 9.3↑ | 1.071 | 10.8 | 0.181 | 209↑ |
| 3-week-1 | 6.07 | 6.57 | 134 | 51 | 653 | 36.3 | 46.4 | 16 | 1 | 0.3 | 2.2 | 2.82 | 0.97 | 0.06 | 0.02 | 33.5↓ | 20.4 | 400 | 8.2 | 27.1 | 9 | 0.588 | 10.7 | 0.177 | 116↑ |
| 2-week-5 | 3.4 | 5.29 | 129 | 54.9 | 701 | 15.5 | 61.4 | 22 | 0.7 | 0.4 | 0.53 | 2.09↓ | 0.75 | 0.02 | 0.01 | 29↓ | 24.4 | 445 | 8.6 | 29.6 | 8.3 | 0.582 | 9.6 | 0.121↓ | 85 |
| 2-week-4 | 3.96 | 5.71 | 134 | 54.9 | 736 | 13 | 62.9 | 22.9 | 0.7 | 0.5 | 0.51 | 2.49↓ | 0.91 | 0.03 | 0.02 | 31.3↓ | 23.5 | 428 | 8.2 | 28.2 | 9.1 | 0.67 | 10.6 | 0.17 | 125↑ |
| 2-week-3 | 7.55 | 5.68 | 135 | 56.8 | 723 | 15.8 | 60.6 | 22.6 | 0.8 | 0.2 | 1.18 | 4.58 | 1.71↑ | 0.06 | 0.02 | 32.3↓ | 23.8 | 418 | 7.3 | 29.6 | 8.5 | 0.615 | 10.1 | 0.144 | 104↑ |
| 2-week-2 | 8.9 | 5.73 | 137 | 55.5 | 820 | 12 | 68.4 | 19.1 | 0.3↓ | 0.2 | 1.06 | 6.09 | 1.7 | 0.03 | 0.02 | 31.8↓ | 23.9 | 431 | 8.2 | 29.6 | 8.9 | 0.73 | 10.6 | 0.163 | 134↑ |
| 2-week-1 | 9.04 | 5.53 | 134 | 52.6 | 863 | 16.8 | 62.1 | 20.5 | 0.4↓ | 0.2 | 1.52 | 5.61 | 1.85↑ | 0.04 | 0.02 | 29.1↓ | 24.2 | 460 | 8.2 | 27.1 | 9.5↑ | 0.82 | 11 | 0.202 | 174↑ |
|  |  |  |  |  |  | 全↑ |  | 全↑ |  |  |  |  |  |  |  |  |  | 全↑ | 全↓ |  |  |  |  |  |  |

Orders of magnitude:

WBC: 10^9^/L, RBC: 10^12^/L, HGB: g/L, MCV: fL, PLT: 10^9^/L, NEU#: 10^9^/L, LYM#: 10^9^/L, MON#: 10^9^/L, EOS#: 10^9^/L, BAS#: 10^9^/L, MCH: pg, MCHC: g/L, RDW-SD: fL, MPV: fL, P-LCC: 10^9^/L.

**
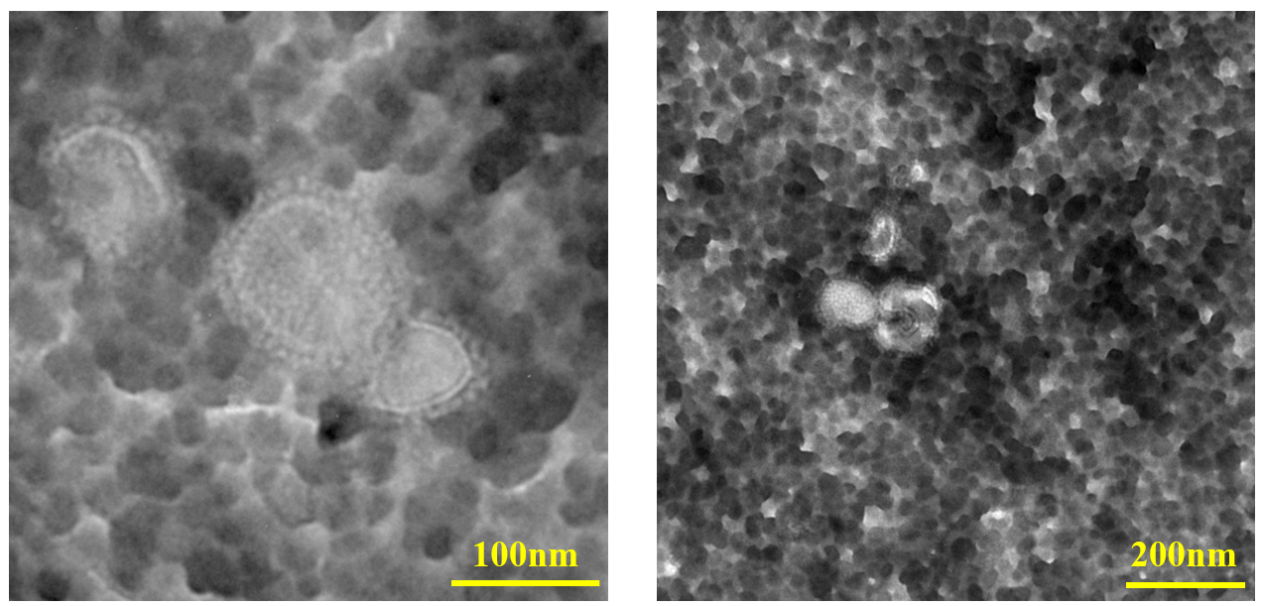
**

**Figure S1.** Untreated viruses show clear and pervasive spikes which can be well differentiated from the medium background.


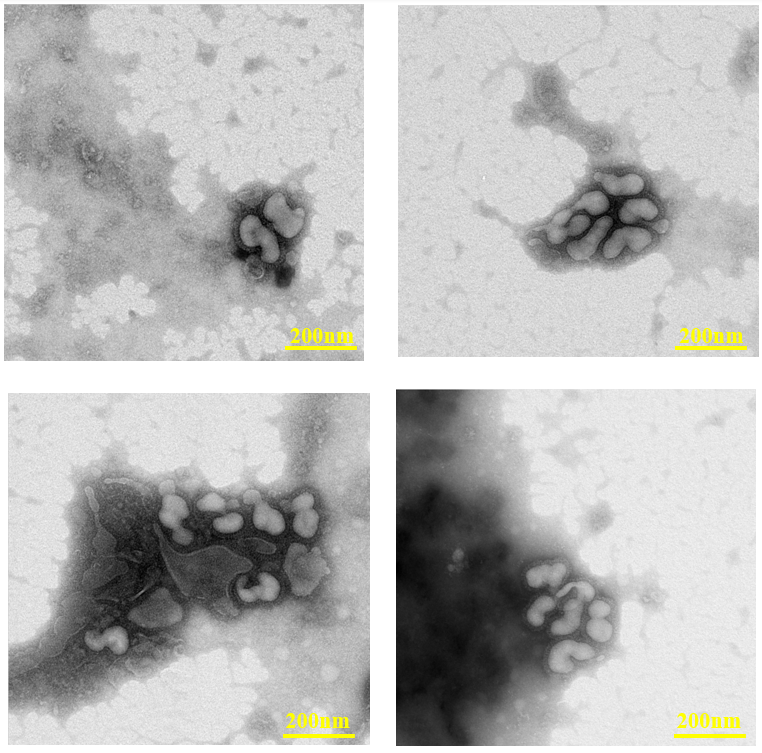


**Figure S2.** Untreated viruses show mixed and multi-shaped virion groups with bright and dark vision areas.


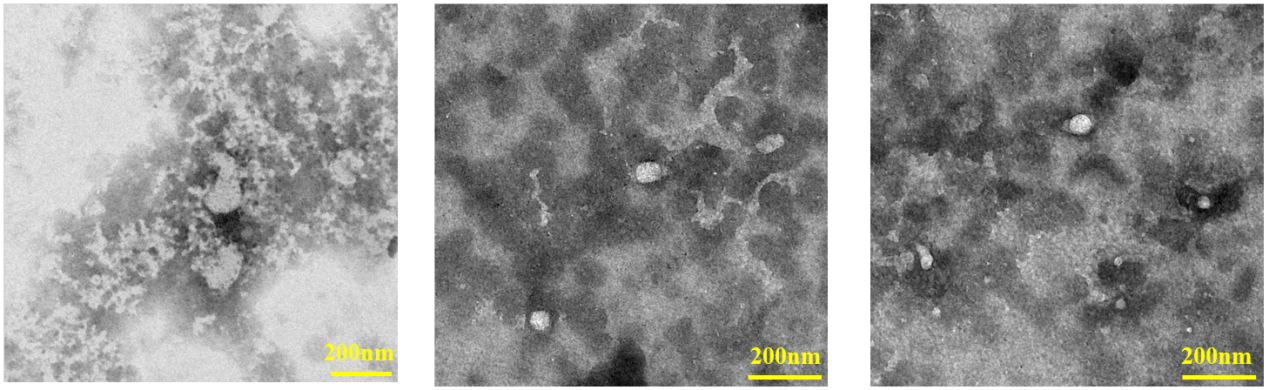


**Figure S3.** Typical spike morphology disappeared and the degree of discrimination from background medium was much reduced. In addition, capsid proteins of groups of coronaviruses have been denatured and clustered with no dark or bright areas.


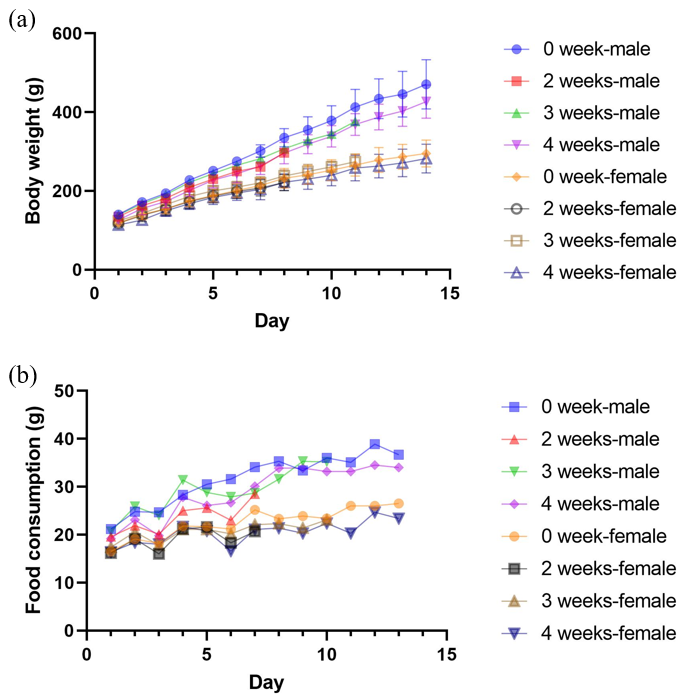


**Figure S4.** Body weight change (a) and Food consumption (b) before (0 weeks) and after 2 / 3 / 4 weeks of plasma exposure.


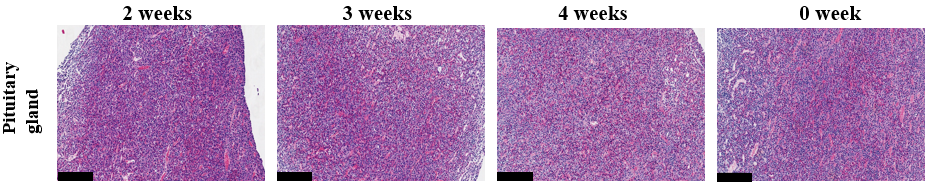


**Figure S5.** HE staining of pituitary body in the endocrine system. Bar:200μm.


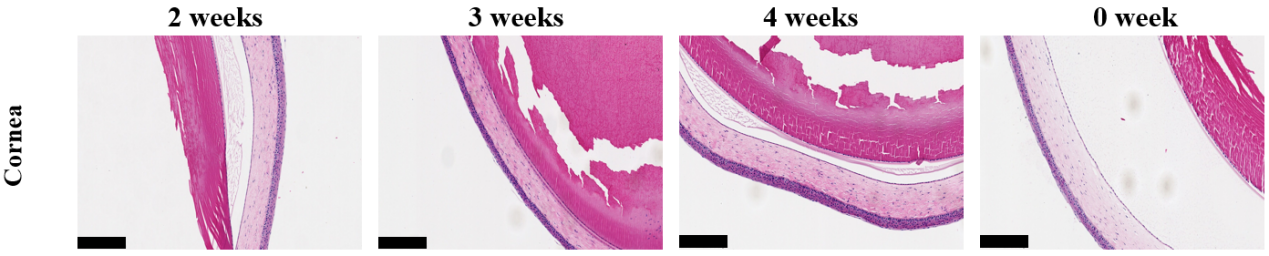


**Figure S6**. HE staining of cornea in the eye of organum sensuum. Bar:200μm.


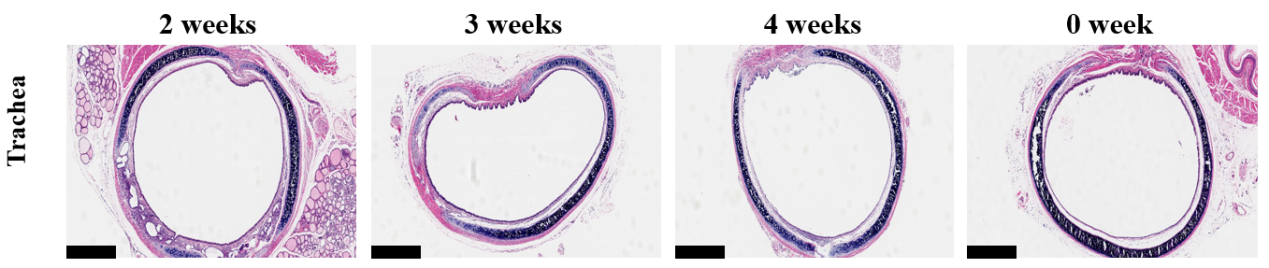


**Figure S7.** HE staining of trachea in the respiration system. Bar:400μm.


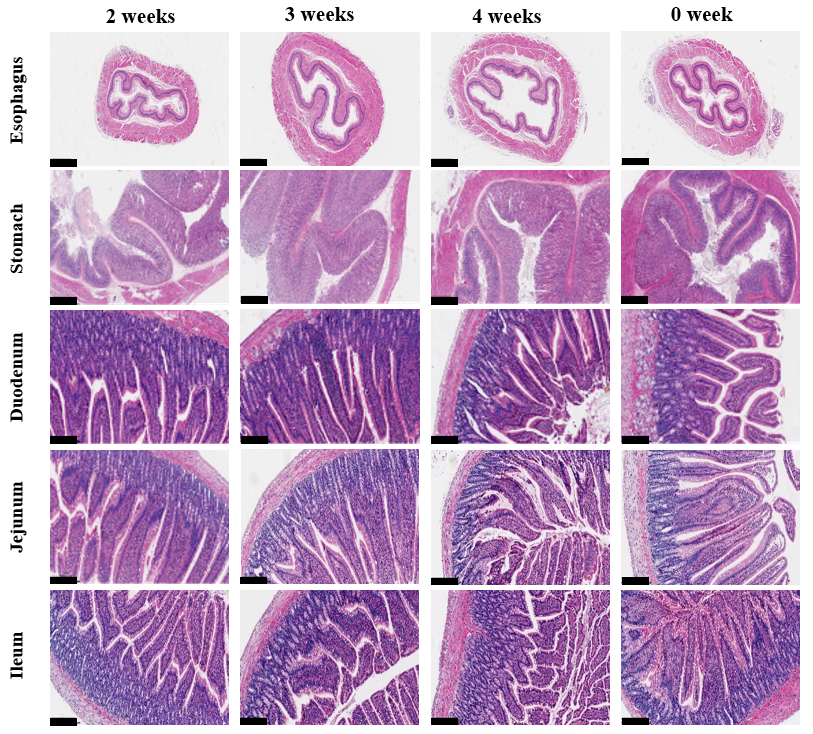


**Figure S8.** HE staining of representative organs in the digestive system. (Bar for Esophagus and Stomach is 400μm and for others is 200μm).


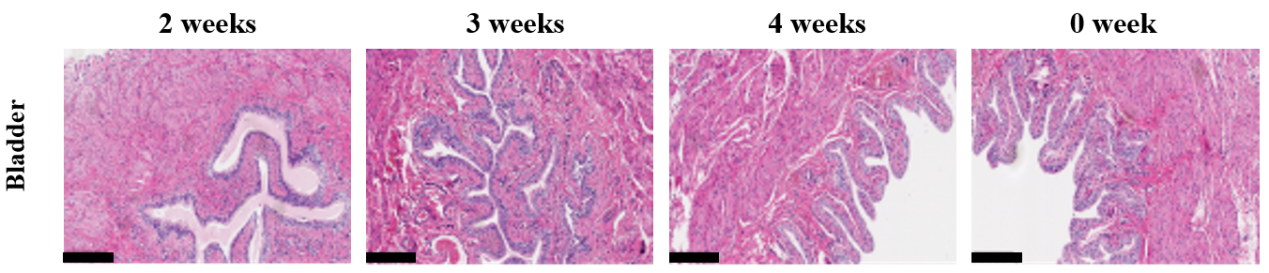


**Figure S9.** HE staining of bladder in the urinary system. Bar:200μm.


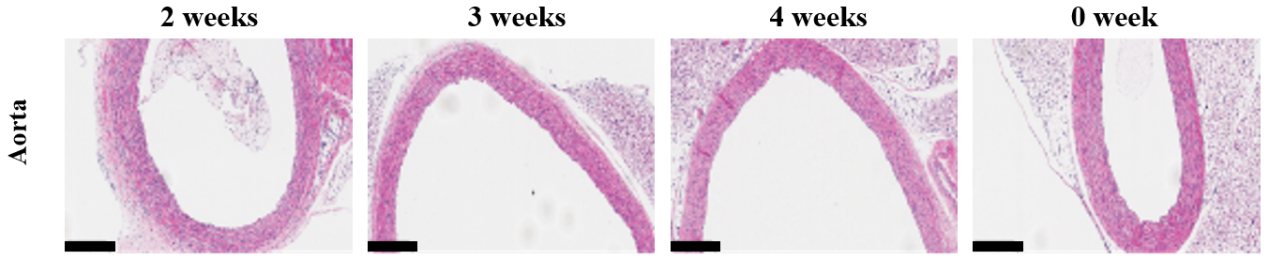


**Figure S10.** HE staining of aorta in the circulatory system. Bar:200μm.


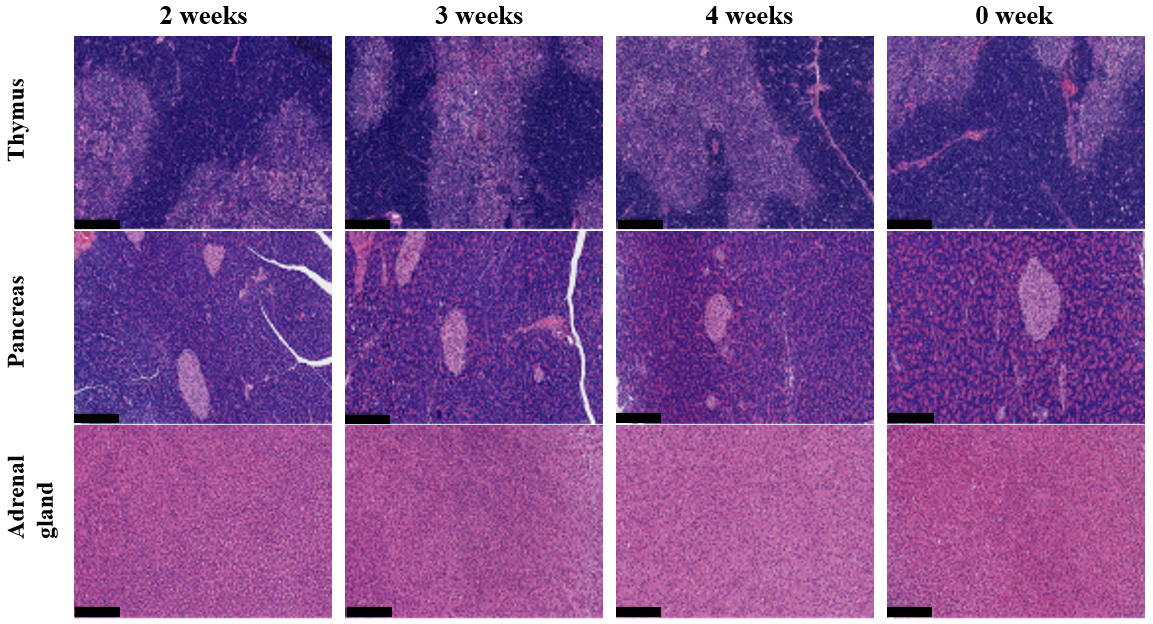


**Figure S11.** HE staining of major organs in the glandular tissue. Bar:200μm.


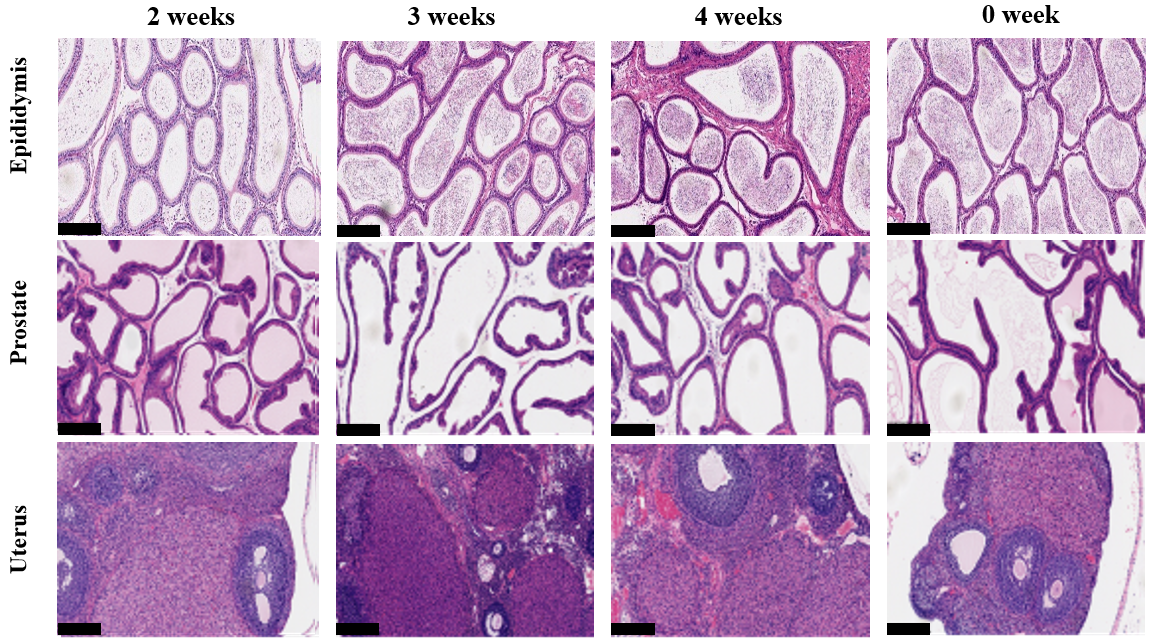


**Figure S12.** HE staining of representative organs in the reproductive system. Bar:200μm.


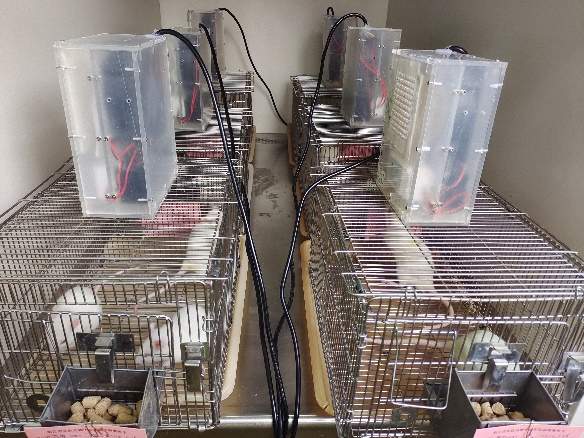


**Figure S13.** Setup for rat exposure to plasma.
